# A suite of global, cross-scale topographic variables for environmental and biodiversity modeling

**DOI:** 10.1038/sdata.2018.40

**Published:** 2018-03-20

**Authors:** Giuseppe Amatulli, Sami Domisch, Mao-Ning Tuanmu, Benoit Parmentier, Ajay Ranipeta, Jeremy Malczyk, Walter Jetz

**Affiliations:** 1 Yale University, Yale School of Forestry & Environmental Studies, New Haven, CT 06511, USA; 2 Yale University, Yale Center for Research Computing, New Haven, CT 06511, USA; 3 Yale University, Center for Science and Social Science Information, New Haven, CT 06511, USA; 4 Yale University, Department of Ecology and Evolutionary Biology, New Haven, CT 06511, USA; 5 Biodiversity Research Center, Academia Sinica, Taipei 11529, Taiwan; 6 University of Maine, Mitchell Center for Sustainability Solutions, 5710 Norman Smith Hall, Orono ME4469, USA; 7 Division of Biology, Imperial College London, Silwood Park Campus, Ascot, Berkshire SL5 7PY, UK

**Keywords:** Ecological modelling, Geomorphology, Hydrogeology

## Abstract

Topographic variation underpins a myriad of patterns and processes in hydrology, climatology, geography and ecology and is key to understanding the variation of life on the planet. A fully standardized and global multivariate product of different terrain features has the potential to support many large-scale research applications, however to date, such datasets are unavailable. Here we used the digital elevation model products of global 250 m GMTED2010 and near-global 90 m SRTM4.1dev to derive a suite of topographic variables: elevation, slope, aspect, eastness, northness, roughness, terrain roughness index, topographic position index, vector ruggedness measure, profile/tangential curvature, first/second order partial derivative, and 10 geomorphological landform classes. We aggregated each variable to 1, 5, 10, 50 and 100 km spatial grains using several aggregation approaches. While a cross-correlation underlines the high similarity of many variables, a more detailed view in four mountain regions reveals local differences, as well as scale variations in the aggregated variables at different spatial grains. All newly-developed variables are available for download at Data Citation 1 and for download and visualization at http://www.earthenv.org/topography.

## Background & Summary

Spatial heterogeneity is an important driver of environmental complexity in a region and influences the sub-regional variation of (i) abiotic factors such as micro/macro climates, soil composition, dynamic processes of the hydrological systems and (ii) biotic factors such as species richness and structure, population complexity, animal movement^1^. Topography as measured by elevation and its derived variables (e.g., slope and aspect), is key for characterizing spatial heterogeneity and the abiotic environment in a given area, subsequently driving hydrological, geomorphological, and biological processes^1,2^. For instance, elevation has numerous dependencies regarding topographic complexity, micro/macro climates or land cover, and can be used to assess biodiversity patterns across the globe by relating species occurrences to environmental factors^3,4^. Besides using the ‘raw’ elevation from a digital elevation model (DEM), a multitude of topographic metrics can be extracted from the DEM to better understand the physical geographic context and landscape properties of a study region^3,5^. For instance, slope and terrain curvatures (defined as a measure of the concavity and convexity, or convergence and divergence) play an important role in catchment-related hydrological responses driving the flow direction, water runoff velocity, water accumulation, soil erosion and soil moisture^6^. Similarly, topographic variation strongly influences the accumulation and heterogeneity of mountain/alpine snow cover^7^ and landslide formation^8^. All these factors regulate the water availability in soil and thus directly influence vegetation moisture, and this constitutes an important element for wildfire risk modeling^9^. Specific terrain attributes may also provide important conditions facilitating the movements and migration of mobile species such as birds^10,11^. Moreover, terrain characteristics may represent vital refugia for species under climate change^12^, a function that likely varies with spatial grain.

Traditionally, DEMs have been derived from aerial photography with stereoscopy or ground surveys. In the last decades, advances in remote sensing technology have contributed to the availability of DEM products with different spatial grains. These products provide gridded information about land surface elevation, where fine spatial grain DEMs (<10 m) derived from laser sensors (e.g., Light Detection and Ranging—LIDAR), and DEMs on medium (10-100 m) to coarse spatial grains (>100 m) are obtained from optical sensors (e.g., Advanced Spaceborne Thermal Emission and Reflection Radiometer—ASTER) and radar sensors (Shuttle Radar Topography Mission—SRTM)^13^. Beside a global cover age at coarse spatial grains, also national and regional DEMs at fine grains are available, such as ArcticDEM (2 m), TanDEM-X (12 m), 3DEP (1 m), CDEM (25 m), AW3D (5 m). Nonetheless, these DEMs are not fully calibrated and their local extension does not allow a global implementation.

Environmental and biological processes depend strongly on the spatial grain of the input variables^14,15^. For instance, to successfully predict species occurrences using species distribution models, the spatial grain of the environmental variables has to match and agree with the sample size and spatial data accuracy, extent of the species data, movement and dispersal range, etc. Coarsening the environmental predictors to, e.g., 50-100 km, may be needed to match coarse atlas data, expert range information, or point records with a high spatial uncertainty^16,17^.

A variety of DEMs elaborated under different projects are currently available and grouped/listed at http://worldgrids.org/doku.php/wiki:dem_sources. However, the calculation and aggregation of elevation-derived variables across spatial grains are typically done on a case-by-case basis. Hence, a coherent and standardized framework for obtaining range-wide, spatially aligned, and ready-to-use topographic variables at different spatial grains is missing. Currently available DEMs may lack the quality required for many applications. For instance, ASTER GDEM contains artifacts and outliers (i.e., abrupt rise—‘humps/bumps’; fall—‘pits’) that require post-processing to remove errors or offsets using additional sources (i.e., SRTM or ICESat laser altimetry)^18^. These anomalies can produce large elevation errors on local scales and impede the use of ASTER GDEM for specific applications. Hydrological applications, for example, require high quality standardized inputs to derive watershed properties that are, in turn, used to track water flows and quality. Similarly, watershed delineation, drainage divide and channel network increasingly rely on digital data from DEMs and derived topography metrics rather than manual or visual interpretation from photography or ground data. In addition, as topography is one of the major driver of local temperature and precipitation patterns, topographic layers (elevation and aspect in particular) are important inputs in many climate layers (e.g., WorldClim http://www.worldclim.org/). Errors or inaccuracies in the inputs can propagate throughout the analyses and derived datasets. Assembling readily available data of high quality is therefore useful in many fields. Of particular relevance for many applications is a global coverage and comparability among different regions. This is emphasized by assessment processes such as those of the Intergovernmental Platform for Biodiversity and Ecosystem Services (IPBES)^19^, where global availability and comparability of data is crucial. The modeling of species distributions worldwide, as targeted in projects such as Map of Life (https://www.mol.org^20^), requires a rich stack of global environmental information, which to date, has only been partially developed in regards to terrain.

To address the lack of readily available topographic variables at a global extent, we calculated a suite of elevation-derived topographic variables based on the 250 m Global Multi-resolution Terrain Elevation Data 2010 dataset^21^ (hereafter ‘GMTED’). As a comparison, we also calculated the most relevant variables using the widely used 90 m Shuttle Radar Topographic Mission^22,23^ void filled dataset SRTM4.1dev^24,25^ (hereafter ‘SRTM’). We resampled (i.e., aggregated) each metric to coarser spatial grains (1, 5, 10, 50, 100 km) using several spatial aggregation approaches to characterize the global topographic heterogeneity (see Aggregated topographic variables session).

In total, we computed 15 continuous variables that characterize the shape of the relief (elevation, slope, aspect sine/cosine, eastness, northness, roughness, terrain ruggedness index, topographic position index, vector ruggedness measure, profile/tangential curvature, first/second order partial derivative) and 1 categorical variable, describing ten major landform classes. The variables (Data Citation 1) are ready to use as input data in various environmental models and analyses, and we provide the code to calculate user-specified variables (e.g., coefficient of variation, range) on custom spatial grains. The newly developed layers are suitable for large-scale environmental analyses and are available for download at Data Citation 1 and for download and visualization at http://www.earthenv.org/topography.

## Methods

For this study, we used the following terminology to define three layer types, each referring to a specific step within the work-flow: source layers are the raw elevation layers from the 250 m GMTED (available as different elevation products, see below) or from the 90 m SRTM dataset. Derived topographic variables are a suite of 15 topographic variables that were calculated from the source layers using moving window analysis. From these derived variables, we obtained the aggregated topographic variables at coarser spatial grains.

### Source layers

Two data sources were used for this work: the 250 m GMTED^21^ and the 90 m SRTM4.1dev^24,25^. GMTED served as the main data set due to its full global extent and having the highest resolution (250 m) available thus far, while SRTM was used for comparing and validating the GMTED-derived variables and for assessing the effect of spatial scale acquisition in regions where the extents of the two data sets overlap (56S—60N latitude).

The GMTED data set (downloaded at http://topotools.cr.usgs.gov/gmted_viewer/) was released in 2010 by the United States Geological Survey (USGS) and the National Geospatial-Intelligence Agency (NGA) globally at three spatial grains 1 km (30 arc-seconds), 500 m (15 arc-seconds) and 250 m (7.5 arc-seconds), with the exception for Greenland and Antarctica, where only 1-km data sets are available. The original GMTED product is a composite product based on several gridded elevation data sources with spatial grains ranging from approximately 30 m to 2 km (1 to 60 arc-seconds, ref. 21). The main source for GMTED is NGAâ€™s SRTM Digital Terrain Elevation Data (http://www2.jpl.nasa.gov/srtm/) at a 1-arc-second resolution (void-filled). Seven additional data sources were used for regions not covered by SRTM, and to fill any remaining holes due to miscellaneous factors such as cloud cover. These included 1) the non-SRTM DTED (NGA) the Canadian Digital Elevation Data (GeoBase—Canada) at two resolutions, 3) the Satellite Pour lâ€™Observation de la Terre Reference 3D (Spot Image—IGN), 4) the National Elevation Dataset for the continental United States and Alaska (USGS), 5) the GEODATA 9 s DEM for Australia (Geoscience Australia), 6) an Antarctica satellite radar and laser altimeter DEM (University of Bristol), and 7) a Greenland satellite radar altimeter DEM (University of Bristol) (see GMTED2010 technical report^21^ for more information). These single DEMs were merged with the global GMTED DEM to a reduced, but standardized spatial grain of 250 m to produce a dataset^21^ with 5 layers that have the values of the mean, median, minimum, maximum, and standard deviation of the native resolution DEMs (1 to 60 arc-seconds) within the aggregated grid cells. The GMTED root-mean-square error (RMSE) ranges are between 25 and 42 m, between 29 and 32 m, and between 26 and 30 m for the 1 km, 500 m and 250 m products, respectively^21^. The 250 m GMTED product does not include Antarctica because the only available DEM is available at a 1 km spatial grain^21,26^. The 250 m GMTED is the only DEM covering the full globe with the highest spatial grain available, consisting of a raster layer of 172800 x 67200 cells.

As a comparison, we also used the 90 m (3 arc-seconds) SRTM4.1dev data produced by the Consultative Group for International Agricultural Research—Consortium (CGIAR) for Spatial Information^24,25^. This product is based on the NASA Shuttle Radar Topographic Mission (SRTM) released in 2003^22,23^. No-data voids relate to water bodies and lakes, shadow effects and areas where insufficient textural information was available for the generation of a DEM. Missing data patches were filled using various methods depending on the availability of other DEM sources (USA—NED 3, Mexico—90 m DM, Canadian Digital Elevation Data Level 1, New Zealand—100 m DEM, Australia GEODATA TOPO 100 k contour data, de Ferrantiâ€™s mountainous areas DEM, CIAT Costa Rica 50 m DEM, Ecuador 90 m DEM; more information regarding the DEMs and and 'holes' in a topographic context is available at http://www.cgiar-csi.org/data/srtm-90m-digital-elevation-database-v4-1). Where available, higher resolution data were used to compute the DEM at 90 m using an interpolation with a vector-based contour and points approach^24,25^. When no additional high resolution DEM was available, an interpolation was performed using the coarser STRM product with the exact method varying according to the size, shape, and topography of the area: 1) kriging and inverse-distance-weighting for small and medium-size holes in flat areas, 2) triangulated irregular network for very flat areas, 3) splines for small and medium holes within areas with complex topography and at high elevations and, 4) advanced splines for large holes with other topography. One limiting factor of the SRTM data is that it only covers low latitude areas between 60N and 58S. In total, the 90 m SRTM4.1dev product consists of a raster of 432000 x 144000 cells. The SRTM data set consists of only one value of altitude per grid cell (as opposed to the GMTED that comes in four different products: median, minimum, maximum, standard deviation).

### Derived topographic variables

**Figure 1:**
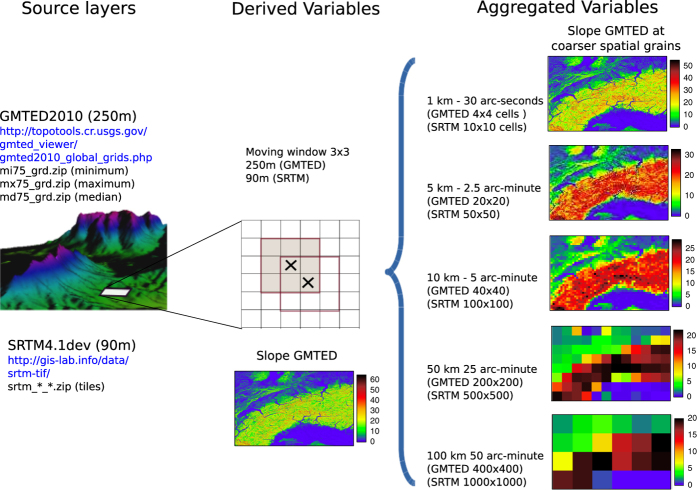
Flowchart describing the suite of topographic variables. The source layers provide the basis for the derived variables, which in turn are then aggregated to coarser spatial grains. First, the derived topographic variables are calculated at the original spatial grain of the source layers (250 m for GMTED and 90 m for SRTM) using a moving window of 3×3 grid cells (gray square). Second, all derived topographic variables are aggregated to coarser spatial grains of 1, 5, 10, 50 and 100 km using a non-overlapping window and various aggregation approaches (see Table 1 for an overview of all newly-developed variables with their aggregation metrics and spatial grain).

As explained in section ‘Source layers’, GMTED is available in the form of several products derived from the different high resolution DEMs, namely minimum, maximum, median, mean and standard deviation (abbreviated in this work and also in the GMTED repository as mi, ma, md, mn, sd, respectively—the same variable abbreviation is used also in Table 1) which can be downloaded from http://topotools.cr.usgs.gov/gmted_viewer/. These GMTED layers were used to calculate the derived topographic variables listed in Table 1. The derived topographic variables were calculated based on 1) the value at each focal grid cell individually, or 2) a set of grid cells in the immediate vicinity of each focal cell as defined by a 3×3 moving window (i.e., the focal cell with eight surrounding cells, see Fig. 1). In the first case, we obtained the mi, ma, and sd elevation variables that were obtained from the source layers 250 m GMTEDmi, 250 m GMTEDma, 250 m GMTEDsd, respectively. These variables are shown in the five first rows of Table 1 (variable name 'elevation'). In the second case, we computed a suite of 16 derived topographic variables, including 15 continuous and 1 categorical variable. The categorical variable has 10 geomorphological classes (see Table 1, last row) that represent the most common landforms. For each of these derived variables, we used the GMTED median elevation (GMTEDmd) as the source. Compared to the mean, the median is less sensitive to outliers and less affected by the potentially skewed distribution of elevation values. Besides the GMTED, the SRTM was also used to calculate a subset of the derived variables as a comparison. For each derived variable (except elevation, see above) we used a 3×3 moving window on the underlying 250 m GMTEDmd source layer and the 90 m SRTM. The full list of variables is shown in Table 1, which provides an overview of the derived topographic variables, the aggregated variables at coarser spatial grains, and the specific source layers (GMTED or SRTM). The third column reports the algorithm used compute each variable and can be used to look up the specific formula used in calculating the derived variables. The value of the derived variables can change slightly from software to software in accordance with the implemented algorithm.

#### Derived topographic continuous variables

**Figure 2:**
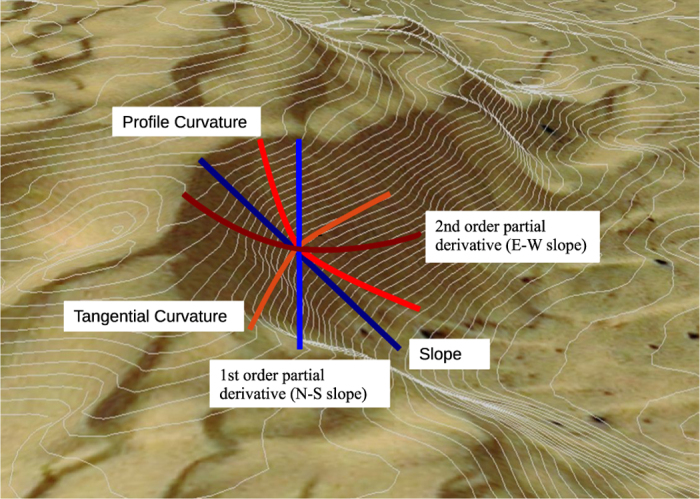
Graphical representation of landform shapes based on slope and curvature. Slope is the rate of change of elevation in the direction of the steepest descent, whereas the second order partial derivative (N-S slope) is the slope in the North-South direction. The profile and tangential curvatures identify concavity and convexity in the direction of the slope, or perpendicular to the slope. The second order partial derivatives (E-W slope) identify the curvature in the East-West direction.

The derived continuous variables include slope, aspect (including sine and cosine values), eastness, northness, four heterogeneity variables (roughness, terrain ruggedness index, topographic position index, vector ruggedness measure), two curvature variables (profile curvature and tangential curvature), and the first and second order partial derivatives of elevation (variables list in Table 1). The partial derivatives describe the rate of change of the slope and curvature while keeping the aspect directions constant (in our case along north-south and east-west directions)^27,28^(Fig. 2). Based on the median elevation of the source layer (GMTEDmd), we first calculated slope and aspect, which are the rate of change of elevation in magnitude and orientation for the steepest descent vector, respectively. The aspect is a circular variable therefore sine and cosine transformation are needed prior to any calculation, including the aggregation to a coarser spatial grain. The sine and cosine of the aspect can be used to emphasize differences in the north/south and east/west exposition. Using aspect and slope, we calculated northness and eastness, which are the sine of the slope, multiplied by the cosine and sine of the aspect, respectively^29^. They provide continuous measures describing the orientation in combination with the slope (i.e., a circular variable is transformed into a continuous one, ranging from -1 to 1). In the north hemisphere, a northness value close to 1 corresponds to a northern exposition on a vertical slope (i.e., a slope exposed to very low amount of solar radiation), while a value close to -1 corresponds to a very steep southern slope, exposed to a high amount of solar radiation.

**Measures of topographic heterogeneity**: Topographic heterogeneity can be described as the variability of elevation values within an area. It can be measured using summary statistics, such as the standard deviation, or specific indices expressing the rate of elevational change in response to changes in location^30^. In this study four indices were calculated to express topographic heterogeneity. Terrain Ruggedness Index (TRI) is the mean of the absolute differences in elevation between a focal cell and its 8 surrounding cells. It quantifies the total elevation change across the 3×3 cells^31^. Flat areas have a value of zero whereas mountain areas with steep ridges have positive values, which can be greater than 2000 m in the Himalaya area. Topographic Position Index (TPI)^32^ is the difference between the elevation of a focal cell and the mean of its 8 surrounding cells. Positive and negative values correspond to ridges and valleys, respectively, while zero values correspond generally to flat areas (with the exception of a special case where a focal cell with a value 5 can have surrounding cells with values of 4×1 and 4×9, resulting in a TPI of 0). Roughness^33^ is expressed as the largest inter-cell difference of a focal cell and its 8 surrounding cells. Vector Ruggedness Measure (VRM)^30^ quantifies terrain ruggedness by measuring the dispersion of vectors orthogonal to the terrain surface. Slope and aspect are decomposed into 3-dimensional vector components (in the x, y, and z directions) using standard trigonometric operators, and by calculating the resultant vector magnitude within a user-specified moving window size (in this study 3×3). The VRM quantifies local variation of slope in the terrain more independently than the TPI and TRI methods^30^. VRM values range from 0-1 in flat and rugged regions, respectively. The four variables TRI, TPI, VRM, and Roughness provide a description of the terrain profile and surface heterogeneity of the landscape surface. In addition to these, the standard deviation of other derived topographic variables can also be used to describe the profile roughness.

**Measures of terrain curvature**: Curvature attributes are based on the change of slope or aspect in a particular direction (graphical representation in Fig. 2 and full variable list in Table 1). The profile curvature measures the change rate of slope along a flow line. It affects the acceleration or deceleration of surface water flow along the surface^28^ and thus influences erosion and deposition of soils^27,34^. In contrast, the tangential curvature measures the change rate perpendicular to the slope gradient and is related to the convergence and divergence of flow across a surface^27,28,34^. The unit of curvature is radians per meter, where positive and negative values indicate convex and concave surfaces, respectively. Concave curvature promotes soil deposition while convex promotes soil erosion. The slope is obtained by mathematical calculation of the first derivative, whereas the curvature is the second derivative. In general, the curvature is highly sensitive to potential errors in the source DEMs because the second derivatives highlight local terrain irregularities^27^.

The first and second order partial derivatives^28^ of the elevation are obtained by keeping the North-South or the East-West direction constant during the slope and profile curvature calculations (Fig. 2). The first order partial derivatives of elevation are useful for distinguishing the slope changes in the North-South from those in the East-West direction. The second order partial derivatives depict the change of profile curvatures (i.e., the convexity or concavity features of the terrain) in the North-South or the East-West direction. The first order identifies the slope change in one direction and is characterized by a smooth surface, whereas the second order shows a salt-and-pepper features that highlight the profile variation on the micro scale.

#### Derived topographic categorical variables

**Figure 3:**
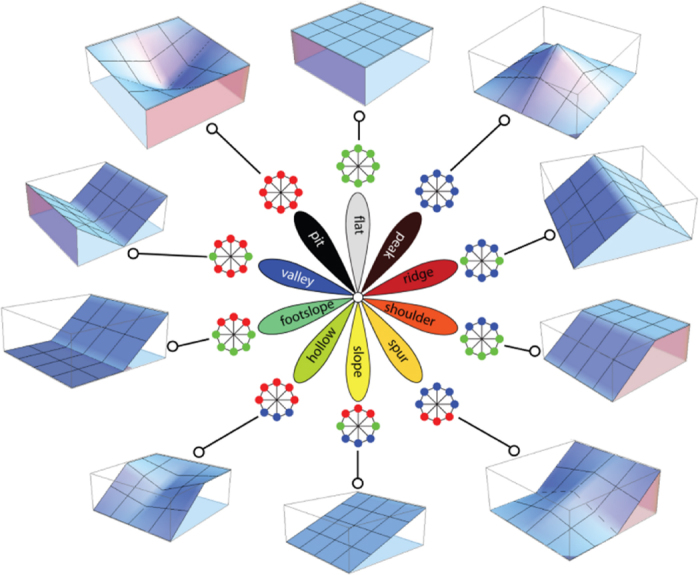
Symbolic representation of the 10 most common landform elements obtained with the GRASS add-on r.geomorphon. (Figure adapted from https://grass.osgeo.org/grass70/manuals/addons/r.geomorphon.html with the author's permission—Dr Jarek Jasiewicz).

**Figure 4:**
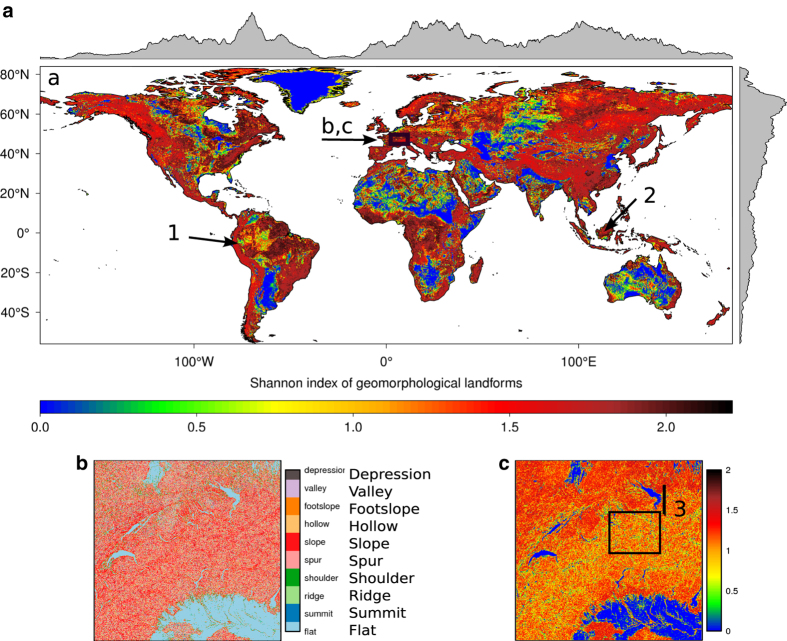
Exemplary map showing the Shannon index of the geomorphological landforms, aggregated to 1 km: a) global view, b) magnification of the 10 underlying geomorphological landforms, c) magnification of the derived Shannon index (the magnification area is part of the Alps and is delineated in plot a). The box in c) represents the study area in Figure 5, and the line 3) identifies the location of the profile depicted in Figure 6. The arrows 1 (Andes) and 2 (Indonesia) drawn in a) point to the location of topographic variable profiles shown in Supplementary Figs 1.

The categorical variable consists of 10 geomorphological landform classes. Geomorphological landform elements are additional topographical features that can be extracted from DEMs using morphometry, which identifies geomorphons (geomorphologic phonotypes)^13^. The output of this method is a raster with grid cell values indicating one of the ten most common landform elements: flat, peak, ridge, shoulder, spur, slope, hollow, footslope, valley, and pit (schematic representation in Fig. 3, Fig. 4b)

### Aggregated topographic variables

The aggregation to coarser spatial grains can be seen as a re-sampling approach that transforms the fine-grain grids to a coarser spatial grain. The derived topographic variables were aggregated to 1, 5, 10, 50 and 100 km spatial grains using different aggregation windows (Fig. 1, dimension of the aggregation window for the five coarser spatial grains were 4×4, 20×20, 40×40, 200×200, 400×400 cells for the GMTED; 10×10, 50×50, 100×100, 500×500, 1000×1000 cells for the SRTM).

**Figure 5:**
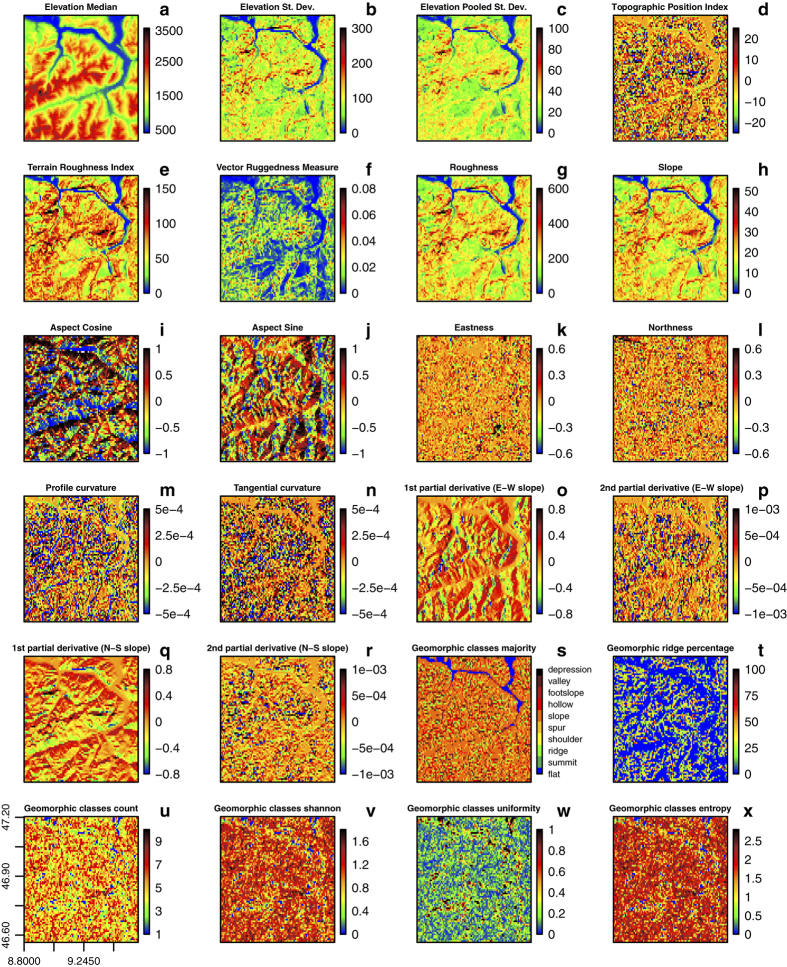
A subset of the derived topographic variables using the 250 m GMTED source layer that have been aggregated to 1 km spatial grain (4×4 cells). The geographic extent (76×72 km) refers to the Alps regions close to Liechtenstein, i.e., the box in Figure 4c. The continuous variables (from ‘a’ to ‘r’) were aggregated using the median value. The categorical variables (Figure 5s-w) were aggregated using six metrics: percent cover of each class (in this case percent of ridge), number of classes, the majority class, Shannon index, entropy and uniformity.

All derived continuous variables were aggregated to the coarser spatial grains (sample overview in Fig. 5) by calculating the median, average, minimum, maximum and standard deviation of the values of grid cells covered by the aggregation window. The categorical variable (geomorphological landform with 10 classes) was aggregated by calculating six metrics: percentage of each class, number of classes, the majority class, the Shannon diversity index, and two gray level co-occurrence matrix (GLCM) based texture metrics (entropy and uniformity)(Fig. 5). The definitions of these six metrics are:

- Percentage of each class: Percent cover of a given landform class within the aggregation window.
- Number of classes: The total number of different landform classes within the aggregation window.
- Majority: The landform class that covers most grid cells of the aggregation window. In case where more than one class is predominant (same number of pixels), a random selection was permitted to choose only one class.
- Shannon Index: A diversity index based on the proportion of grid cells covered by the landform types within the aggregation window. Higher values indicate more landform types and/or types having more similar proportions within an aggregation window^35^(Figs. 4a and 4c).
- Entropy: a GLCM-based second-order image texture metric which quantifies the disorderliness of the spatial arrangement of pixel values (landform types in this case) within the aggregation window. A higher value indicates that different landform types are distributed more randomly within an aggregated window^36^.
- Uniformity: a GLCM-based second-order image texture metric which quantifies the uniformity of pixel values (landform types in this case) within an aggregation window. It is also called the angular second moment^36^. A higher value indicates that different landform types are distributed more regularly within an aggregated window.

### Ancillary user-specified derived topographic variables

From the derived topographic variables, other user-specified and customized variables can be created at different spatial grains. For example, the coefficient of variation (cv) of the profile curvature at 1 km resolution can be calculated as

pcurv_1KMcv_GMTEDmd.tif=pcurv_1KMmn_GMTEDmd.tif/ pcurv_1KMsd_GMTEDmd.tif

The range (rg) of the slope at 5 km can be computed as

slope_5KMrg_GMTEDmd.tif=slope_5KMma_GMTEDmd.tif- slope_5KMmi_GMTEDmd.tif

### Tools and work flow

All calculations were processed in parallel using open-source software at the Yale Center for Research Computing at Yale University. To allow parallel and distributed processing of our work-flow, we split the GMTED raster data set into 4 x 2 tiles (43200 x 33600 cells each) and the SRTM raster into 36 x 12 tiles (12000 x 12000 cells). In both cases, the neighboring tiles overlapped with 2 grid cells (thus GMTED: 43204 x 33604 cells; SRTM: 12004 x 12004 cells). To accelerate the computation, the global DEM was sent to each single computational node and each single tile was processed by one CPU core. The neighboring tiles overlapped with 2 grid cells to avoid any border artifacts during the calculation of the derived variables. After the variable calculation, these duplicate cells were then removed when merging all tiles to global maps.

The derived topographic variables were computed with the Geospatial Data Abstraction Library (GDAL, http://www.gdal.org/, version number 1.10.0)^37^ and with the Geographic Resources Analysis Support System software (GRASS, https://grass.osgeo.org/, version number 7.0.0)^28,38^. Table 1 reports the GDAL or GRASS commands used for each derived variable calculation. GDAL and GRASS provide fast and flexible computation features and functions for raster-based scripted automated workflows, and allow processing of very large data sets due to efficient algorithms and memory management. In the context of our work, they allowed on-the-fly calculations of true geodesic distances in a latitude-longitude coordinate reference system, enabling direct work in the native projection system of both the GMTED and SRTM products, i.e., the World Geodetic System 1984 (EPSG:WGS84).

To aggregate the derived continuous variables to coarser spatial grains, we used the Processing Kernel for geospatial data (pktools, http://pktools.nongnu.org, version number 2.6.3)^39^. The *pkfilter* function with the —*down* flag option allowed us to run aggregations for user-specified defined window sizes while the —*filter* flag option allowed selection of the aggregation approach (mn, mi, mn, md, sd). The aggregation for the derived categorical variable was performed with *pkfilter* for the percent count and majority. For the Shannon index, entropy and uniformity, a customized python script was implemented. We provided example code at http://spatial-ecology.net/dokuwiki/doku.php?id=wiki:grass:grasstopovar for users to calculate and aggregate custom variables at specific spatial grains. Moreover, on the same page we provide example R codes to download a specific study area given a bounding box extent.

## Data Records

We provide aggregated topographic variables derived from the 250 m GMTED at the spatial grains of 1, 5, 10, 50, and 100 km. For a subset of variables (see variable list in Table 1) we also provide the aggregated variables from the SRTM source layer. All variables are available for download at Data Citation 1. A visualization of the layers is given online at www.earthenv.org/topography, where users can browse a subset of variables.

Each variable, aggregated at different spatial grains, is distributed at a global extent (56S—84N latitude—excluding Antarctica) in a compressed GeoTiff file format in the WGS84 coordinate reference system (EPSG:4326 code). All layers were stored as floating point (Float32 datatype) for maximum precision, allowing the computation of custom variables (e.g., coefficient of variation). In addition the derived topographic variables at the original 250 m GMTED or 90 m SRTM can be recomputed on demand.

## Technical Validation

In this section we compared the differences and correlations between the derived topographic variables and also among the aggregated topographic variables at different spatial grains. In particular we explored the (i) divergence of the most common variables obtained starting from two source layers (GMTED and SRT) aggregated at the different spatial grain, (ii) correlation among the topographic variables that characterized the terrain heterogeneity, and (iii) correlation among the topographic variables that describe the relief forms and shapes. The correlation analysis highlights the multicollinearity among variables and can be useful to select uncorrelated independent variables in regression models to ensure appropriate inference^40^.

### Topographic variable profile

**Figure 6:**
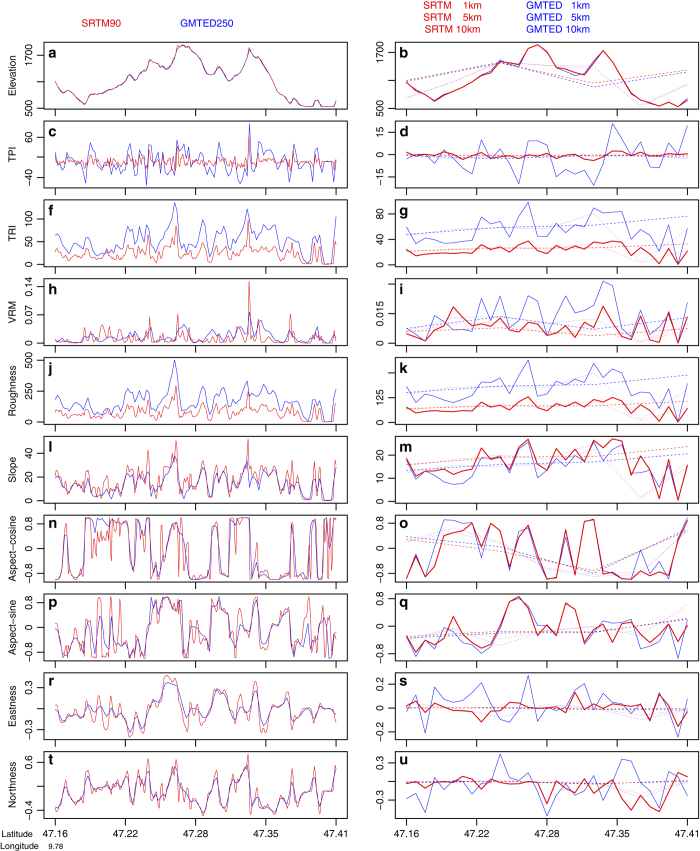
Profile of the topographic variables for a transect line of 28 km in the Alps region close to Liechtenstein. Geographic location depicted in Figure 2c. On the left: variable values obtained from 250 m GMTED and 90 m SRTM, on the right: variable values after a median aggregation at 1, 5 and 10 km (for other profiles in different areas see Supplementary Fig. 1).

To evaluate the effects of the aggregation to coarser spatial grains we compared the profiles of the variables along an elevation gradient (Fig. 6 left side). Roughness, TPI and TRI variables derived from the 250 m GMTED showed a higher variance (Fig. 6c,f,h, fluctuation of the blue lines) compared to those derived from the 90 m SRTM (red lines). This was expected as any adjacent 90 m-cells in the SRTM data would have a smaller elevation gradient relative to the focal cell, compared to the GMTED with a larger cell size of 250 m. In fact the three variables were computed as a function of the difference of a central pixel and its surrounding cell. Thus the GMTED derived variables have higher values with a higher fluctuation. Likewise, VRM values derived from the SRTM showed a higher sensitivity to local variations in the input data (e.g., red line peaks in Fig. 6h) and to DEM resolution compared to the VRM derived from the GMTED. The aspect and slope derived variables based on SRTM also showed a higher variability than those obtained by the GMTED (Fig. 6l,n,p,r,t).

We also evaluated and compared the effects of aggregation for the 1, 5 and 10 km grid cell size (Fig. 6 right side and for 1-5-10-50-100 km in Supplementary Fig. 1). In general, GMTED and SRTM derived variables showed a higher variability at 1-5 km spatial grains, and tended to show similar spatial trends at coarser grains (>10 km, dashed lines, see also Supplementary Fig. 1—right side). The aggregated variables based on GMTED displayed more fluctuation than those derived from the SRTM. This was due to the fact that adjacent cells of SRTM had less vertical difference than adjacent cells of GMTED, therefore producing smaller values of heterogeneity also when aggregated (median) to a coarser spatial grain.

### Heterogeneity comparison

**Figure 7:**
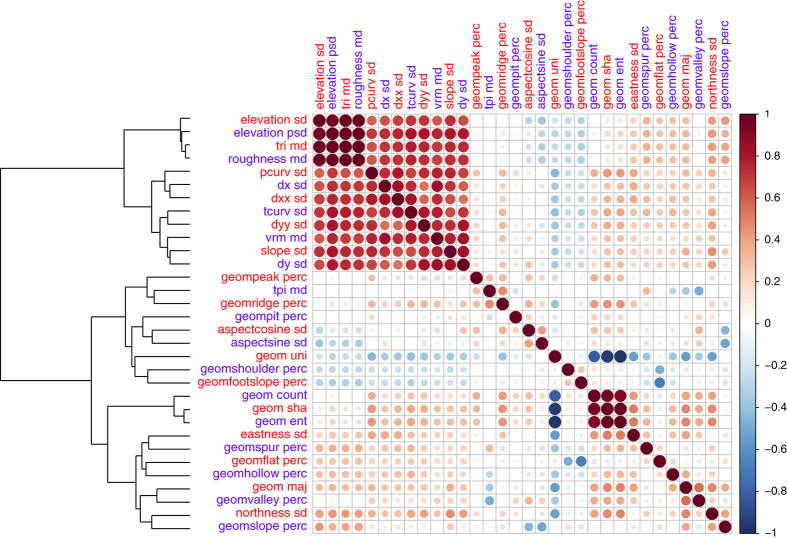
Correlation matrix plot (32×32 variables) for the GMTED derived topographic variables (aggregated to 1 km spatial grain), describing heterogeneity and roughness in the Alps region depicted in Figure 2b (2400×1600 cells). Variable name and aggregation approach abbreviation are reported in Table 1. The scale bar reports Pearson's positive (red) and negative (blue) correlation coefficients. Circles in the plots have different sizes according to the absolute values of correlation coefficients. The GMTED derived topographic variables are labeled in red and blue to better distinguish the column and row of the plot and are ordered according to a hierarchical clustering on the values obtained from 200,000 1-km pixels randomly selected from the Alps region.

Topographic heterogeneity, or terrain roughness, was defined as the spatial variability in elevation and also by the feature and relative distribution of the different landform elements within a given area. In the first case, the heterogeneity was described by the four heterogeneity variables (TRI, TPI, VRM and roughness) or their aggregated medians at coarser spatial grains. In addition, the standard deviation of the other derived variables (e.g., standard deviation of the slope) also provided information about the amount of topographic variation or dispersion at a given level of aggregation (from 1 to 100 km), describing indirectly the spatial-vertical heterogeneity. In the second case, the five metrics of the geomorphological landforms (percent, Shannon index, entropy, uniformity, count) described terrain shape heterogeneity. Fig. 7 shows the cross-correlation and hierarchical clustering dendrogram among all aggregated topographic variables that express directly or indirectly the terrain heterogeneity. The dendrogram is useful to see how ‘far or close’ two variables are, and how they can used to depict different features of the terrain.

### Topographic shapes comparison

**Figure 8:**
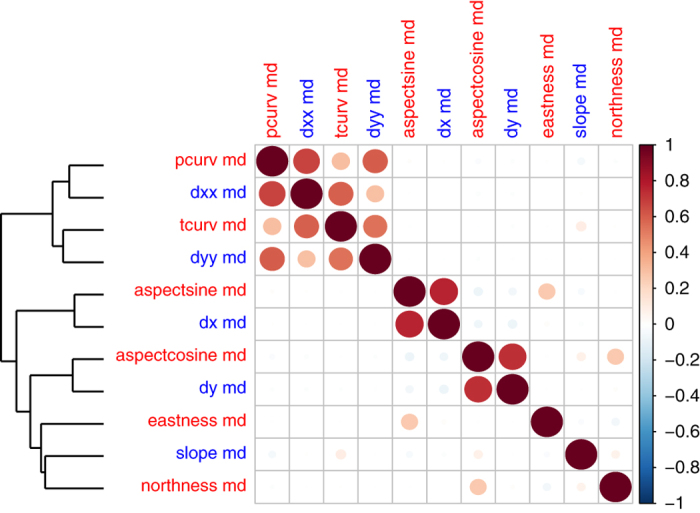
Correlation matrix plot (11×11 variables) for the GMTED derived topographic variables (aggregated at 1 km spatial grains), describing the relief shape in terms of slope, aspect and curvatures in the Alps region depicted in Figure 2b (2400×1600 cells). Variable name and aggregation approach abbreviation are reported in Table 1. The scale bar reports Pearson's positive (red) and negative (blue) correlation coefficients. Circles in the plots have different sizes according to the absolute values of correlation coefficients. The GMTED derived topographic variables are labeled in red and blue to better distinguish the column and row of the plot and are ordered according to a hierarchical clustering on the values obtained from 200,000 1 km pixels randomly selected from the Alps region.

The topographic shapes of a relief in terms of slope, aspect and curvature was described by a group of variables (aspect cosine, aspect sine, slope, eastness, northness, profile and tangential curvature, first and second order partial derivatives) that emphasized one of the terrain shapes or described the features of two shapes combined. The former case was the second order partial derivative in the East-West slope direction that combined curvature information in a particular slope direction. To understand the relationship and collinearity among those variables, we depicted a correlation plot and the relative hierarchical clustering in Fig. 8, which identifies two main branches. The upper branch includes all the topographic variables that describe profile curvatures, whereas the lower branch includes all the variables that are indirectly derived from aspect and slope. In particular, the curvature variables are strongly correlated in the upper left part of Fig. 8.

### Quality control and data limitation

A visual inspection of the 250 m and 1 km GMTED derived variables revealed a few artifacts and issues. We documented striping and/or blocky artifacts visible especially in Eurasia (above 60N latitude, see Supplementary Fig. 4 showing an area of 840×700 km in Siberia). The artifacts were visible in all the GMTED derived variables with different level of magnitudes. These artifacts were due to the production method of the non-SRTM DTED dataset (see the note by the GMTED developer^21^) and could not be corrected prior to processing the topographic variables. We found that the Canadian area covered by the CDED did not present the same artifacts. Nonetheless a vertical gap was visible between the GMTED derived from SRTM, and the GMTED derived from the CDED (Northwest Territory in Canada). Artifacts and vertical gaps were more visible and evident in flat areas where the pixel value fluctuation was due to data acquisition methods rather than effective altitudinal variations. Beside the artifacts visible in the GMTED-derived variables, we found that the SRTM-derived variables also presented some anomalies (Supplementary Fig. 5). The SRTM artifacts were due to void filling procedures performed during the SRTM4.1dev^24^ production workflow. In the same area, the GMTED derived variables did not present such anomalies, due to a better filling procedure mixed with a smoothed effect of the 250 m resolution^21^.

Despite these limitations the GMTED is still the only available DEM covering the full globe at 250 m spatial grain. We advise users to note the presence of artifacts in the area above 60N latitude and carefully check for possible effects in their own analyses and results.

## Usage Notes

The newly-developed topographic variables (Data Citation 1) have a wide variety of potential applications in ecology, biogeography, conservation and biodiversity science, land use and land cover change, physical geography, hydrological and climate sciences. For instance, the variables are ready to use for topographical analyses, or as predictors for assessing species distributions across multiple spatial grains over large spatial extents in a standardized framework^41–43^. For hydrological applications, the newly developed variables can be used in the context of flow and erosion modeling at different spatial grains.

More specifically, slope and curvature can serve as useful input variables in erosion and hydrological models, as well as in species distribution models to depict micro-habitats that are mainly driven by the shape of the fine-grain topography. In climate science, some of these topographic layers can be used as covariates in the interpolation of temperature and precipitation for local, regional or global layers. Topographic variables are also useful for land use change modeling applications that involve the determination of suitability and locations for different land use types (e.g., agriculture, residential, recreation). While the correlation analysis (Figs. 7 and 8) shows how several of the derived variables are closely related over a large spatial extent, we highlight that over smaller spatial extents, an accurate description of the local topography is better provided by a mixture of different variables (Fig. 5, e.g., TRI combined with the eastness and profile curvature).

We provide several aggregation levels to be used for scale-dependent sensitivity analyses and models, or as a basis during the implementation of a given algorithm. From a computational point of view, the variables with a coarser spatial grain (e.g., 50 and 100 km) can be used during the testing of code or workflow development as they are less computationally demanding than the variables at 250 m or 1 km spatial grain. In the spirit of reproducible research we provide the scripting procedure (http://spatial-ecology.net/dokuwiki/doku.php?id=wiki:grass:grasstopovar) to calculate custom topographic variables, and to aggregate these variables to specific spatial grain. The procedure can also be used to recompute the derived topographic variables at the original resolution (250 m GMTED and 90 m SRTM4.1dev). Moreover, we also show how to load the GeoTiff format in R and how to make a colorful map. Users are thus encouraged to use their own DEM layers and implement the scripting procedure to generate the desired topographic variables at different spatial grain by changing the parameter of aggregation. Additional layers regarding other topographic variables (e.g., topographic wetness index) are currently under development, and we encourage potential users to contact authors about future products updates.

The suite of newly-developed topographic variables at 1 km spatial grain can be useful in combination with other 1 km global layers, such as the consensus land-cover layers^44^, the global habitat heterogeneity metrics^44^ and freshwater environmental variables^45^ for species distribution modeling^46^ or environmental analyses and models in general. This combination with other environmental layers (e.g., solar radiation, relative humidity, and soil moisture) representing ecological mechanisms offer broad potential, but was beyond the scope of our study. Overall, we expect that the current terrain layers will benefit a range of uses in geography, geology, hydrology, ecology and biogeography, and especially applications requiring large spatial extents, a rich stack of different terrain variables, and/or versatility to address multiple spatial grains^47–49^.

## Additional information

**How to cite this article**: Amatulli, G. *et al*. A suite of global, cross-scale topographic variables for environmental and biodiversity modeling. *Sci. Data* 5:180040 doi: 10.1038/sdata.2018.40 (2018).

**Publisher**’**s note**: Springer Nature remains neutral with regard to jurisdictional claims in published maps and institutional affiliations.

## Supplementary Material



Supplementary Information

## Figures and Tables

**Table 1:** List of the derived and aggregated topographic variables.

**Variable name**	**Variable abbreviation**	**Command used**	**Aggregation 1, 5, 10, 50, 100** **km (software used: pkfilter)**	**Source layers**
Elevation	elevation		mi	Minimum (mi)
Elevation	elevation		ma	Maximum (ma)
Elevation	elevation		mn, sd	Mean (mn)
Elevation	elevation		*mi, ma,* md, sd	Median (md)
Elevation	elevation		sd, psd	Standard Deviation (sd)
Roughness	roughness	gdaldem	mi, ma, mn, md, sd	Median (md)
Terrain Ruggedness Index	tri	gdaldem	mi, ma, mn, md, sd	Median (md)
Topographic Position Index	tpi	gdaldem	mi, ma, mn, md, sd	Median (md)
Vector Ruggedness Measure	vrm	r.vector.ruggedness.py	mi, ma, mn, md, sd	Median (md)
Aspect Cosine	aspectcosine	gdaldem gdal_calc.py	mi, ma, mn, md, sd	Median (md)
Aspect Sine	aspectsine	gdaldem gdal_calc.py	mi, ma, mn, md, sd	Median (md)
Slope	slope	gdaldem gdal_calc.py	mi, ma, mn, md, sd	Median (md)
Eastness	eastness	gdaldem gdal_calc.py	mi, ma, mn, md, sd	Median (md)
Northness	northness	gdaldem gdal_calc.py	mi, ma, mn, md, sd	Median (md)
Profile curvature	pcurv	r.slope.aspect	mi, ma, mn, md, sd	Median (md)
Tangential curvature	tcurv	r.slope.aspect	mi, ma, mn, md, sd	Median (md)
First order partial derivative (E-W slope)	dx	r.slope.aspect	mi, ma, mn, md, sd	Median (md)
First order partial derivative (N-S slope)	dy	r.slope.aspect	mi, ma, mn, md, sd	Median (md)
Second order partial derivative (E-W slope)	dxx	r.slope.aspect	mi, ma, mn, md, sd	Median (md)
Second order partial derivative (N-S slope)	dyy	r.slope.aspect	mi, ma, mn, md, sd	Median (md)
Geomorphological landforms (10 types: flat, peak, ridge, shoulder, spur, slope, hollow, footslope, valley, pit)	geom	r.geomorphon	perc, count, maj, sha, ent, uni	Median (md)
All topographic derived variables are based on the 250 m GMTED and/or 90 m SRTM, using a 3×3 cell moving window analysis on (see Figure 1). The abbreviations in the third column correspond to the aggregation approach for each of the five spatial grains (1, 5, 10, 50, 100 km): minimum (mi), maximum (ma), median (md), mean (mn), standard deviation (sd), pooled standard deviation (psd), number of classes (count), percent prevalence of each class (perc), majority or most abundant class (maj), Shannon index (sha), entropy (ent), uniformity (uni). The different text styles of the aggregation column identify the DEM source: normal text style refers to variables derived from GMTED; text in *italic* refers to variables derived only from SRTM; underlined text refers to variables derived from both GMTED and SRTM. Final layer names are a combination of: variable abbreviation _ aggregation resolution—relative aggregation metric _ DEM source layers (with the underneath aggregation for the GMTED). For example, tri_1KMmd_GMTEDmd.tif, aspectcosine_5KMsd_SRTM.tif. All data are stored in Data Citation 1.				
