# Supplementary material for: A suite of global, cross-scale topographic variables for environmental and biodiversity modeling
Source: Sci Data. 2018 Mar 20;5:180040. doi: 10.1038/sdata.2018.40 (PMC5859920; doi:10.1038/sdata.2018.40)
Supplement: Supplementary Information [file sdata201840-s2.pdf]

SCIENTIFIC DATA

Overleaf

Supplementary Information

Figure 1. Profile of the topographic variables for a transect line for Alps region: .... page 2

Figure 2. Profile of the topographic variables for a transect line for Amazonas region: .... page 3

Figure 3. Profile of the topographic variables for a transect line for Borneo region: .... page 4

Figure 4. GMTED 250 m topographic variables showing artifacts for Siberian regions: .... page 5

Figure 5. SRTM 90 m and GMTED 250 m Vector Ruggedness Measure for Mexico region: .... page 6

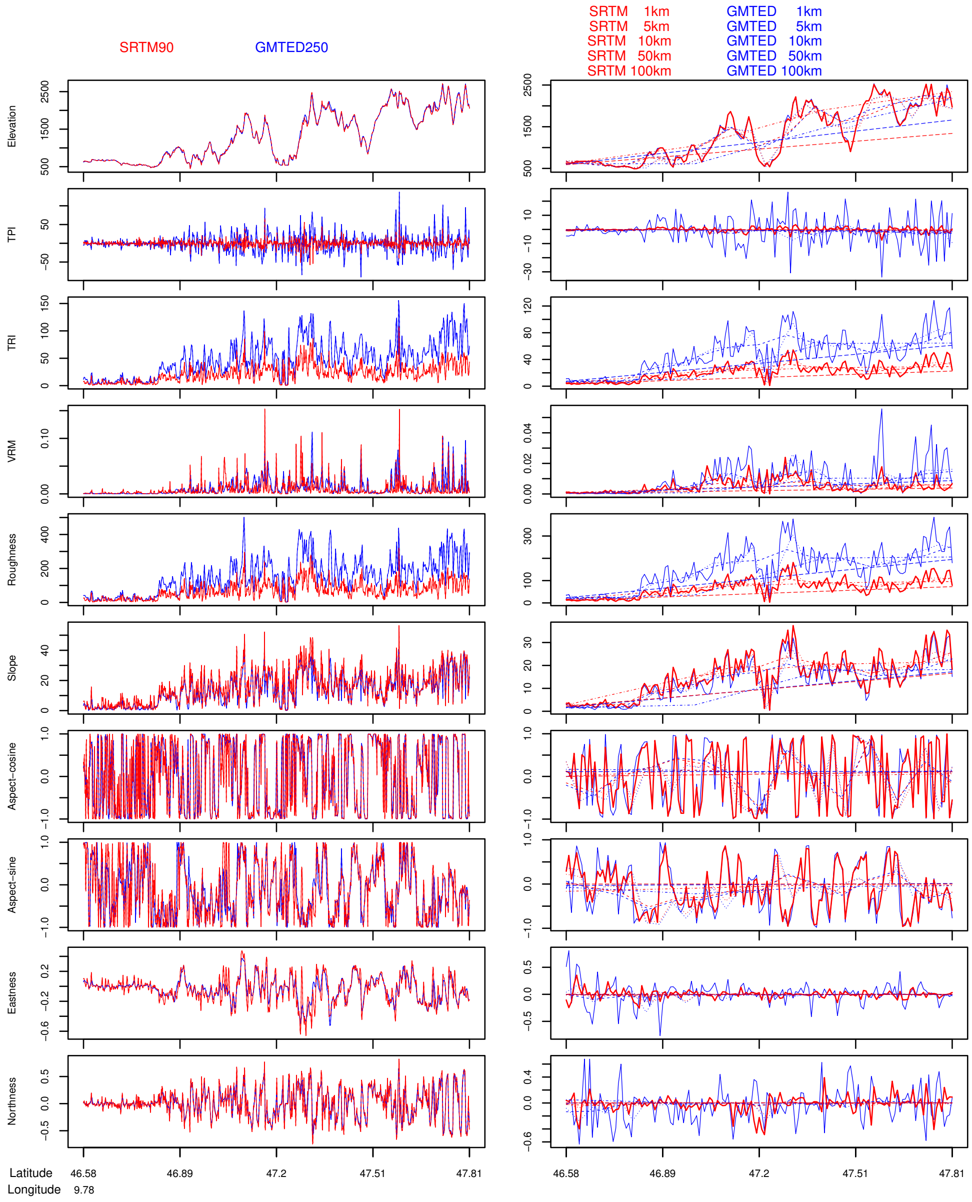

Figure 1: Profile of the topographic variables for a transect line of 30km in the Alps region close to Liechtenstein. Geographic location depicted in Figure 2c. On the left: variable values obtained from 250m GMTED and 90m SRTM, on the right: variable values after a median aggregation at 1, 5, 10, 50, 100 km.

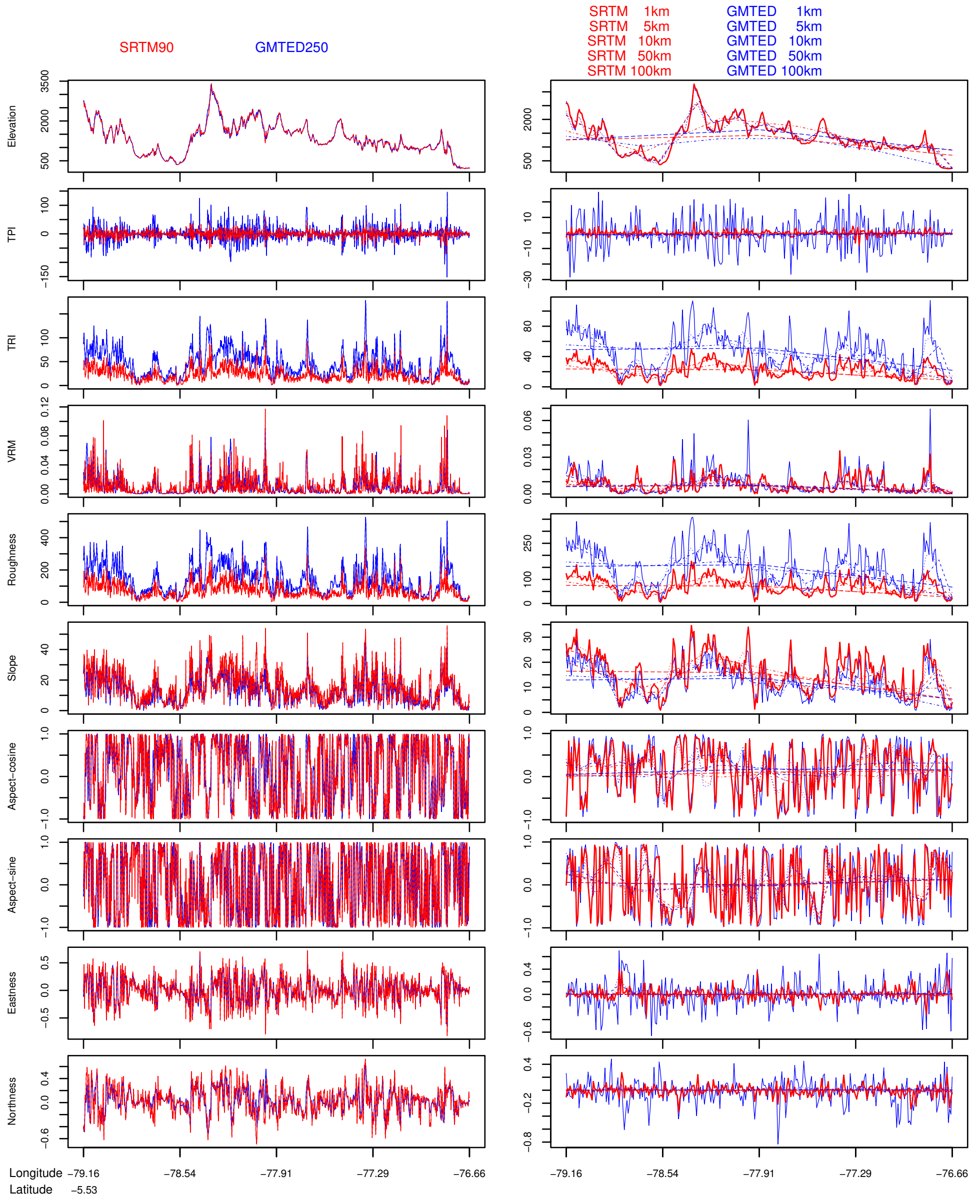

Figure 2: Profile of the topographic variables for a transect line of 277 km in the Amazonas Region of Peru. Geographic location depicted in Figure 2c. On the left: variable values obtained from 250m GMTED and 90m SRTM, on the right: variable values after a median aggregation at 1, 5, 10, 50, 100 km.

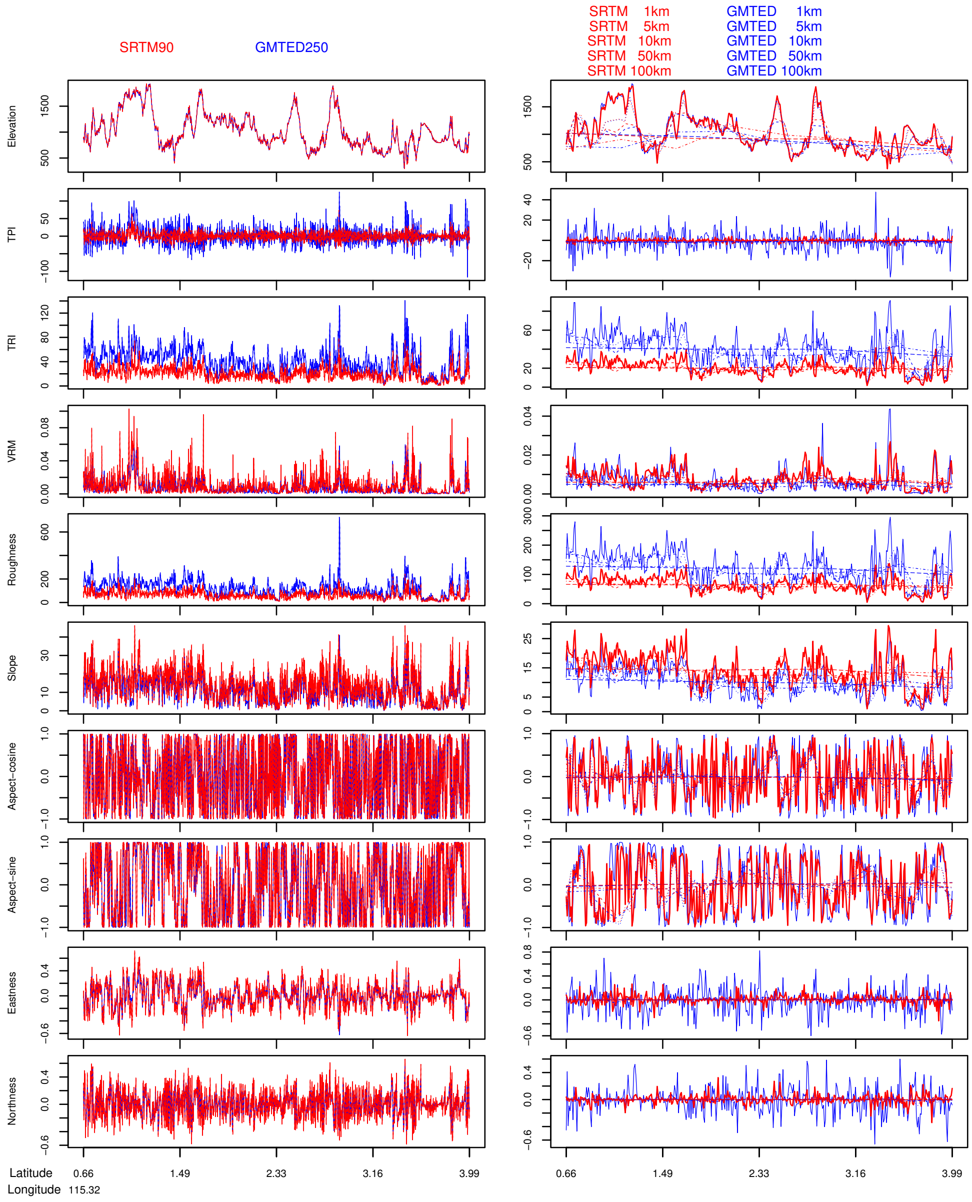

Figure 3: Profile of the topographic variables for a transect line of 370 km in the Borneo Region. Geographic location depicted in Figure 2c. On the left: variable values obtained from 250m GMTED and 90m SRTM, on the right: variable values after a median aggregation at 1, 5, 10, 50, 100 km.

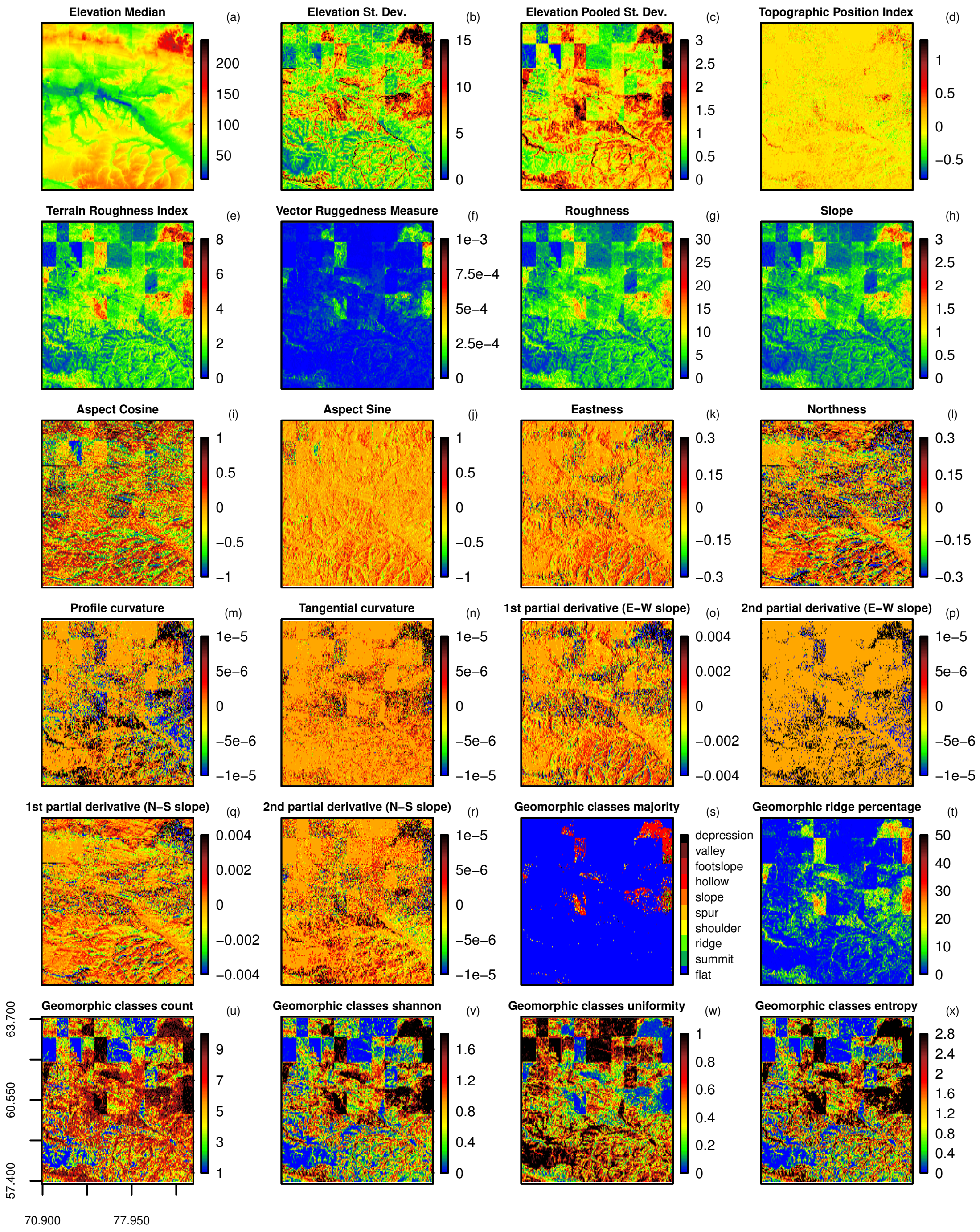

Figure 4: A subset of the derived topographic variables using the 250m GMTED source layer which have been aggregated to 5 km spatial grain (20x20 cells). The geographic extent (6.4° x 14.2°) refers to the Siberian regions where artifacts are present due the data acquisition problems of the underlying 250m GMTED data sources. The artifacts are present only in the area above 60°N latitude and only in the non-SRTM DTED dataset (Euroasia zone)

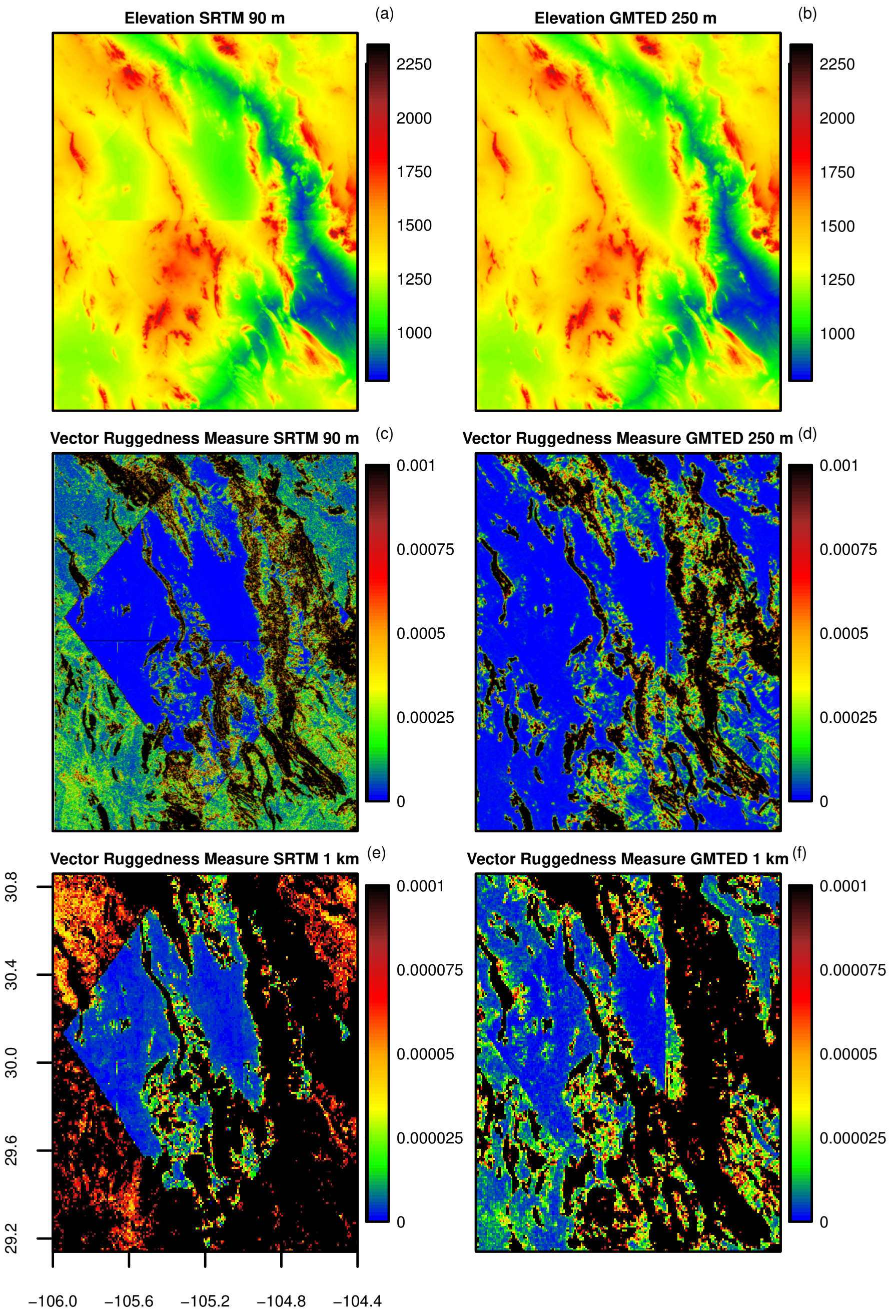

Figure 5: A subset of SRTM 90 m (a) and GMTED 250 m source layers (b) and the derived Vector Ruggedness Measure (VRM) variable. VRM at the original spatial grain (c,d) and aggregated to a 1 km spatial grain (median; e,f). The geographic extent (2.6° x 1.6°) refers to a desert area in Mexico close to the border of USA. The artifacts are mainly visible in the SRTM source layers (left side), where a horizontal line is clearly visible in the middle of the figure (a). The artifacts are also present in the derived topographic variables (c) and aggregated variables (e). In contrast, the artifact is not visible in the GMTED source layer due to a different void filling process. Consequently, the variables derived from the GMTED source layers seem to be more homogeneous across than those derived from SRTM.
